# Teratocarcinosarcoma of the cheek: a case report

**DOI:** 10.1093/jscr/rjac169

**Published:** 2022-04-12

**Authors:** Mohamed Reda El Ochi, Amine Essaoudi, Abderrahim El Ktaibi, Amal Damiri, Hafsa Chahdi, Mohamed Oukabli

**Affiliations:** Department of Pathology, Mohammed V Military Hospital, Rabat, Morocco; Faculty of Medicine and Pharmacy of Rabat, Mohammed V University, Rabat, Morocco; Department of Pathology, Mohammed V Military Hospital, Rabat, Morocco; Faculty of Medicine and Pharmacy of Rabat, Mohammed V University, Rabat, Morocco; Department of Pathology, Mohammed V Military Hospital, Rabat, Morocco; Faculty of Medicine and Pharmacy of Rabat, Mohammed V University, Rabat, Morocco; Department of Pathology, Mohammed V Military Hospital, Rabat, Morocco; Faculty of Medicine and Pharmacy of Rabat, Mohammed V University, Rabat, Morocco; Department of Pathology, Mohammed V Military Hospital, Rabat, Morocco; Faculty of Medicine and Pharmacy of Rabat, Mohammed V University, Rabat, Morocco; Department of Pathology, Mohammed V Military Hospital, Rabat, Morocco; Faculty of Medicine and Pharmacy of Rabat, Mohammed V University, Rabat, Morocco

## Abstract

Teratocarcinosarcoma is a rare and aggressive malignant tumor of uncertain histogenesis. It presents <1% of all cancers and ~3% of malignant tumors of the head and neck. It arises commonly from the nasal cavity and paranasal sinuses. To the best of our knowledge, only one case has been reported in the oral cavity. A 46-year-old woman presented with 3-week history of a rapidly growing tumor in the inner side of the left cheek. Physical examination revealed an ulcerating mass measuring 4 × 3.5 cm. An excisional biopsy was performed. Histological analysis revealed a teratocarcinosrcoma. The patient was treated by combined chemotherapy and radiation therapy. No recurrence was noted 6 months after treatment. The prognosis is poor.

## INTRODUCTION

Teratocarcinosarcoma (TCS) is an uncommon and aggressive malignant tumor of uncertain histogenesis [1], which is mainly located in the sinonasal tract [2].This neoplasm is often misdiagnosed and characterized by variable microscopic features consisting of an intimate mixture of benign and malignant epithelial, mesenchymal and neuronal elements [3–5]. It is accepted that this tumor develops from primitive embryonic tissues or pluripotential cells that have remained sequestrated in the sinonasal tract [1, 2]. Since the first description in 1984 by Heffner and Hyams [3], several cases have been reported, with only one case reported in the oral cavity [2]. Here, we report an exceptional case involving the left cheek and we discuss the clinicopathological features and the differential diagnosis.

## CASE REPORT

**Figure 1:**
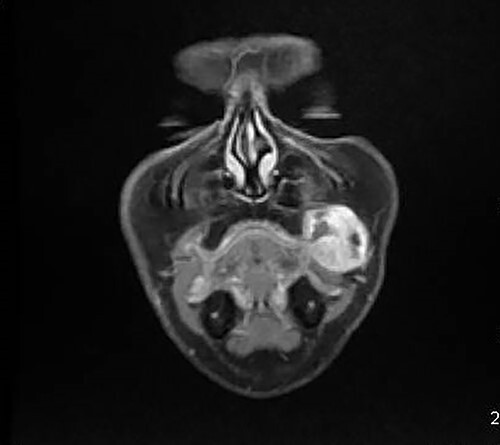
MRI showing the tumor of the left cheek with contrast enhanced.

**Figure 2:**
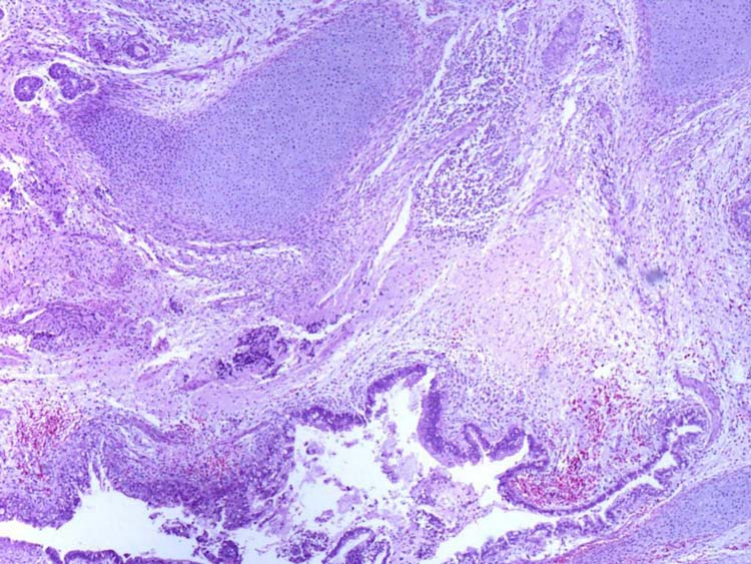
Mature glandular components with cartilage (magnification at ×40).

**Figure 3:**
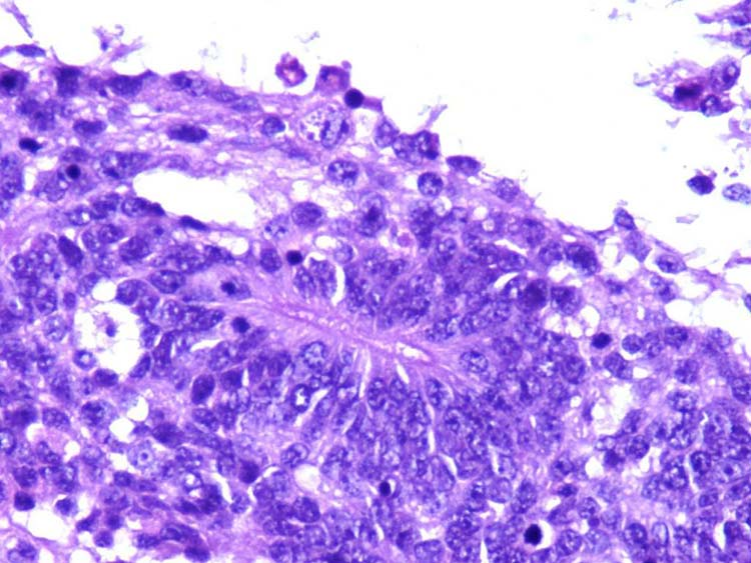
Photomicrograph of primitive neuroectodermal component with neural rosettes (magnification at ×400).

**Figure 4:**
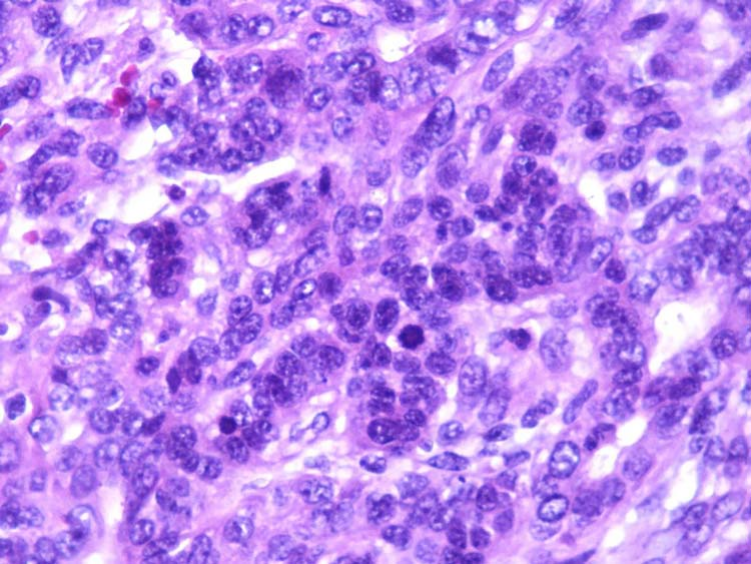
Photomicrograph of carcinomatous component (magnification at ×400).

A 46-year-old woman, without clinical antecedent, presented with a tumor in the inner side of the left cheek which had rapidly grown for 3 weeks. Physical examination revealed an ulcerating mass measuring 4 × 3.5 cm, well-circumscribed, fleshly, with a white surface. There was submandibular lymphadenopathy, ranging in dimension from 1 to 1.5 cm in diameter. Magnetic resonance imaging (MRI) scan revealed the tumor of the cheek without mandibular bone infiltration or sinonasal involvement (Fig. 1). Chest computed tomography and abdominal ultrasound scan were unremarkable. An excisional biopsy was performed. Pathological examination showed a neoplastic proliferation composed of mixture of mature benign epithelial components, such as intestinal type, respiratory and squamous epithelia, and mature mesenchymal components, such as muscular tissue and cartilage (Fig. 2) and neuroglial tissue. In addition, immature and malignant tumor components were found such as small blue tumor cells resembling primitive neuroectodermal tumor (Fig. 3), adenocarcinoma and sarcoma with muscular and cartilaginous differentiation (Fig. 4). This tumor infiltrates the skeletal muscle with free margins. Immunohistochemically, epithelial components were positive for pankeratin; mesenchymal components showed desmin, myogenin and S-100 positivity and the primitive neuroectodermal tumor component was positive for NSE, CD99, GFAP, synaptophysin and focally for chromogranin. SALL4 was negative. The resected submandibular lymph nodes were devoid of malignant infiltrate. The diagnosis of TCS was established and the patient was treated by combined chemotherapy (cisplatine +5 fluorouracil) and radiation therapy. No recurrence was noted 6 months after treatment.

## DISCUSSION

TCS is an exceptional aggressive malignant tumor, presenting <1% of all cancers and ~3% of malignant tumors of the head and neck [6]. It arises commonly from the nasal cavity and paranasal sinuses with only one case reported in the oral cavity (floor of the month) [2, 7–9]. Majority of the cases were observed in adult population with male to female ratio of about 4:1 [6]. Histologically, it shows polymorphous features with mixture of epithelial, mesenchymal and neuroectodermal tissues. The epithelial components include areas of adenocarcinoma, squamous carcinoma, glandular structures lined by benign epithelium and areas of nonkeratinizing squamous epithelium [10]. The fetal-appearing clear cell squamous epithelium and the presence of organoid structures are important keys for diagnosis [3, 10]. The mesenchymal components may include benign and malignant fibroblasts or myofibroblasts, cartilage, osteogenic tissue or skeletal muscle [2, 10]. The teratoid components include organoid structures or neural rosettes and neurofibrillary matrix [10].

On immunohistochemistry, the epithelial cells are cytokeratin- and epithelial membrane antigen-positive; mesenchymal cells may be positive for smooth muscle actin, desmin, myogenin and S-100; teratoid cells are positive for neuron-specific enolase, CD99, chromogranin, synaptophysin and glial fibrillary acidic protein [10].

The most important differential diagnosis is carcinosarcoma, which is characterized by the presence of a single mesenchymal component and the absence of neuroectodermal tissue [2]. This pitfall can be ruled out by adequate tissue sampling and, in some cases, only by complete excision of the tumor [1]. Another differential diagnosis is immature malignant teratoma which is positive for SALL4 [6].

The histogenesis of TCS is uncertain and it is hypothesized that this tumor arises from primitive embryonic tissue or immature pluripotential cells [2, 5].

The treatment is based on surgery followed by radiation therapy and sometimes by chemotherapy [1, 2, 6].

This tumor has an aggressive behavior with mean survival reported to be 1.7 years and 60% mortality rate within 3 years [3].

In summary, TCS is a rare neoplasm, morphologically heterogeneous, composed of benign and malignant epithelial, mesenchymal and neuronal components. Adequate sampling is needed for correct diagnosis. Complete surgical resection and adjuvant therapy seem to be the treatment of choice. This management, despite the aggressive behavior of the tumor, improves the prognosis.

## CONFLICT OF INTEREST STATEMENT

None declared.

## FUNDING

None.

## AUTHORS’ CONTRIBUTIONS

All authors read and approved the final manuscript.

## ETHICS APPROVAL AND CONSENT TO PARTICIPATE

Obtained.

## CONSENT FOR PUBLICATION

Obtained.
